# Overdose risk environment for people who use drugs in New Jersey: Imagining possible points of intervention for harm reduction practitioners

**DOI:** 10.21203/rs.3.rs-5919998/v1

**Published:** 2025-01-31

**Authors:** Nora Sullivan, Michael Enich, Rachel Flumo, Stephanie Campos, Netanya Flores, Jenna Mellor, Caitlin O’Neill, Amesika N. Nyaku

**Affiliations:** Rutgers University School of Social Work; New Jersey Harm Reduction Coalition; Cooper University Healthcare; John Jay College of Criminal Justice; Temple University School of Podiatric Medicine; New Jersey Harm Reduction Coalition; New Jersey Harm Reduction Coalition; Northwestern University

**Keywords:** risk environment, enabling environment, harm reduction, overdose

## Abstract

**Background:**

The Risk Environment Framework is widely utilized theoretical framework for understanding the landscape of harm for people who use drugs (PWUD). This study sought to understand factors contributing to risk of overdose for PWUD in New Jersey. Understanding these factors can lead to improved policy interventions, programmatic targets, and a shared understanding that overdose risk is impacted by larger societal forces influencing PWUD.

**Methods:**

Using a community based participatory design model, this study conducted 30 semi-structured, in-depth interviews with PWUD and naloxone distributors in New Brunswick and Newark, New Jersey from February to November of 2022. Thematic analysis was performed using a collaborative analytical approach.

**Results:**

Risk factors for overdose fell into all four categories of Rhodes’s Risk Environment Framework – physical, social, economic, and policy. Many factors overlapped in multiple categories, and most factors had elements existing at both the macro and micro levels.

**Conclusions:**

Interventions supporting PWUD should see overdose risk as an environmental, structural consideration, and be constructed to address comprehensive risks, rather than directing themselves exclusively at the individual level. Factors contributing to risk at the macro level included systemic and institutional concerns and stigma toward PWUD. At the micro level, mental health, substance use behaviors, treatment and recovery, and trauma were cited as potential risk factors.

## 1. INTRODUCTION

People who use drugs (PWUD) are internationally understood to be at higher risk of adverse health outcomes, including higher disease prevalence of infectious diseases (such as HIV, Hepatitis B and C, and tuberculosis), mental health conditions, cardiovascular conditions, and respiratory conditions. They are also at markedly higher risk of experiencing both fatal and non-fatal overdoses (1). In the United States, provisional data available at the time of this study from the Centers for Disease Control and Prevention estimated over 103,000 people have died of a drug overdose with over 75,000 of them likely attributable to opioids (2). Negative stigma towards PWUD and experienced discrimination contribute to these poor physical health outcomes, while alienation and discrimination are also associated with poorer mental health (3).

Multiple theoretical frameworks have emerged to understand the degree to which social and structural factors contribute to both infectious disease and overdose risk for PWUD. The risk environment framework for understanding drug-related harm was useful in its initial application because it moved away from individualistic interventions to reduce HIV risk to consider the macro and micro physical, social, economic and policy environment that contribute to HIV risk (4). Subsequent iterations of the risk environment have focused on the intersectional nature of these social and structural factors that contribute to overdose risk (5). Across applications, these studies highlight how structural factors like gendered power dynamics, the physical location of drug use, economic conditions PWUD must engage with for survival, and drug abstinence policies all contribute to overdose risk, among many other factors (4, 5).

There are applications of the risk environments in geographic areas that give researchers and advocates specific insight into how local cultural, political, and social factors contribute to overdose or infectious disease risk (6–8). These studies highlight potential commonalities across cultural and geographic contexts, such as the influence lack of housing contributes to potential overdose risk (7, 8), or how over-policing leads to stigma and criminalization of drug use (6–8). These studies also reveal important local factors that uniquely contribute to overdose risk, such as place-specific legal requirements around mandated substance use treatment (6), extremely heightened local fears around HIV transmission (8), and the complex interplay between local ability to access housing within a specific social welfare system (7).

Studies have employed the risk environment framework or similar theoretical orientations to reveal potential points of intervention in their communities (9). However, no work has specifically examined the risk environment in urban New Jersey where racial and ethnic disparities in overdose deaths are concentrated (10, 11, 12). Additionally, while some research has critically analyzed naloxone distribution in the context of local risk environment (13), further research is needed to assess the impact of the local risk environment on the feasibility of interventions by harm reduction practitioners in the context of Overdose Education and Naloxone Distribution (OEND).

This study’s goal is to elucidate the overdose risk environment in two urban areas in New Jersey and to provide actionable steps for practitioners to address these risks. From qualitative interviews with people who use drugs and naloxone distributors, we describe the local overdose risk environments and how they imagine what practitioners need to address in their programming to align with the priorities of people who use drugs.

## 2. METHODS

### 2.1 STUDY DESIGN

This study was designed using pragmatic inquiry. Pragmatism as a theoretical orientation uses straightforward questions to offer practical and useful insights on real-world issues (14). Under this orientation, the study used community-based participatory research methods (CBPR) to inform all parts of the research process. CBPR engages affected community members and encourages them to participate in research processes, and is useful in unpacking persistent health disparities within marginalized communities (15, 16). University-based researchers partnered with a local harm reduction organization to inform research design, and this organization, community groups and interviewees all participated in research analysis, member checking, and dissemination of results. The study was reviewed and approved by a university Institutional Review Board.

### 2.2 Sample Recruitment and Eligibility

Participants were identified through three organizations in two urban cities in New Jersey and by respondent-driven sampling. NJ was chosen as an appropriate study site based on the state’s diverse population and growing harm reduction efforts. Participants were over 18 years old, English speaking, and self-identified as people who used drugs and/or naloxone distributors. Original sample stratification aimed to interview 30 individuals and over-sample PWUD in each area by 2:1; in practice, participants overlapped between categories.

Partner organizations distributed flyers on regular outreach or distributed to participants believed to be eligible. Participants were also given referral coupons to distribute to peers they believed might be willing to participate. A total of 30 individuals completed interviews and were compensated $100 for their initial 60–90 minute interview, $25/referral for a maximum of 2 referrals, and an additional $50 for additional contributions by reviewing interview results. Participant sample demographics are described in Table 1.

### 2.3 DATA COLLECTION

A semi-structured interview guide was developed in collaboration with the primary partner organization and asked questions about experience with opioid overdose, perception of the local overdose risk environment, and experiences engaging with peers who use drugs. Questions were driven largely by previous work examining the risk environment (4). Full interview guide is available in the supplemental materials.

The research team (comprised of at least one senior research team member and usually two graduate student team members) conducted in-depth, semi-structured interviews between February and July of 2022. Interviews were collaborative but would be led by one team member while others present took notes. Most often they were in person, but three interviews with naloxone distributors were conducted via Zoom. All but one interview was recorded and transcribed by an outside company. All analysis was done using Dedoose version 9.0.54 (SocioCultural Research Consultants, Manhattan Beach, California, USA). On average, interviews were 47 minutes long. Within 18 interviews, we determined code saturation had been reached (or that the identified themes would reflect the diversity of all possible themes). Through an iterative process of code discussion at team meetings and an interim analysis, we determined that meaning saturation occurred within 28 interviews (17).

### 2.4 ANALYSIS

Qualitative analysis was deductive and inductive. A codebook was developed with deductive sensitizing themes derived from the risk environment framework and interview guide. To pilot, the codebook was applied on four interviews with three team members coding the transcript and revised to include inductive themes and clarifications. For subsequent transcripts, coding was completed by two members of the research team and then collaboratively audited by a senior coder in order to reach coding consensus for each interview. Cross-case analysis grouped together responses of similar sensitizing themes and divergent perspectives for additional inductive coding (14).

Fifteen participants completed follow-up interviews reviewing study results, and feedback from these sessions were integrated into inductive themes. This contributed to the study’s rigor, along with member checking from community partners, triangulation from study staff members, negative case analysis and consensus coding among staff. After results were confirmed, stakeholder meetings were held with community partners to distribute knowledge back to the community.

## 3. RESULTS

### 3.1 Micro and Macro Risk Environment

Study participants acknowledged risk factors at both micro and macro levels that contributed to their risk of overdose and other drug-related harms. Research on the micro risk environment has examined the role of risk perception and behavior related to social norms, rules and values, as well as peer groups and social settings (4). Literature on macro risk environment factors describe public and legal environments, economic factors and identity-based factors related to categories of oppression such as gender or race, and social inequality. Many factors were applicable at both micro and macro levels due to the fluid nature of these structures in peoples’ lives. For instance, a risk factor operating at the systemic level may also impact PWUD at an institutional or individual level. People also spoke to envisioned solutions that would meaningfully address those risks.

Following Rhode’s risk environment framework (4), the findings from this study include elements of all four of his proposed environmental risk types: physical, social, economic, and policy. Findings are categorized accordingly below, with attention paid to whether they occurred at the micro or macro levels–with factors sometimes occurring simultaneously at both levels and within multiple environment types. Major themes with additional illustrative quotes are provided in Table 2.

### 3.2 Physical Environment

Multiple factors in the physical environment impact participants’ sense of increased risk of overdose at the micro level and macro level, with some falling into both categories.

#### Micro risk factors

One micro-level risk factor that participants reported was the risk of using drugs alone. Participants discussed that overdose is often a taboo subject and the secrecy of drug use leads to overdose. This creates potentially deadly barriers to prevention:

In my experience with people I’ve lost, they had to hide it…And we know that’s how people die…People die in those shadows, you know?

(520, ND, former PWUD)

Ongoing stigma and denial of drug misuse was a reason that many PWUD are forced to use in isolated physical environments. Often, this was seen as an issue in wealthier areas:

There are counties, there are towns where we say hey, can we come in, and they’re like no thank you, you know, we don’t have a drug problem here. And it’s not true, right? It’s across, you know, look at the stats, look at the numbers.

(718, ND)

#### Macro risk factors

Participants were asked to share their perceptions of how and where overdoses occur in their community and the way the community responds to these overdoses. Participants knew places in their communities that were hotspots for overdose. Sometimes these places felt inescapable:

You go downtown. A lot of them be out there overdosing. They be, they be on, they be on a tilt out there, leaning…So a lot of people be sitting there overdosing. They said, on my block, they said somebody was there dead for 3 days and they just left them on the steps off of overdosing.

(199, PWUD)

Inadequate housing was another concern related to the physical environment for overdose risk. Participants expressed a desire for increased housing availability for unhoused PWUD. A lack of housing contributed to stress driving substance use, as well as a lack of safe space to use responsibly that made it difficult to make progress towards individual goals:

I wish they find shelter. And maybe that be a wake-up call for them. You have a roof over your head, a key to put in your door.

(200, PWUD)

#### Overlapping micro and macro risk factors

Participants also shared that many times it was necessary to physically remove oneself from an environment that encouraged substance use. Additionally, participants spoke of the risk of exiting a treatment environment and returning to a location with reduced support, and the high likelihood of overdosing at that time. This combines micro-level elements related to treatment and recovery with the environmental challenges that may encourage a return to use:

So if an individual’s mandated, you know, into treatment or into detox, or even incarceration, right, when they come out, if they relapse, they’re not going to start back at the beginning again. So they’re going to, you know, relapse and use up that last amount. But they don’t realize their tolerance level has dropped.

(718, ND)

#### Proposed solutions to physical environment risk factors

In addition to housing, people provided many physical solutions for overdose prevention, such as mobile harm reduction centers, naloxone vending machines, and providing naloxone at convenience stores. Some proposed community resource centers where people could access needed resources and community support. Finally, several participants spoke in favor of overdose prevention centers where people who use substances could use safely and spend time:

Those spaces where you can just be out loud and you can say I’m using drugs today, can I hang out here, because I don’t have anywhere else to be.

(520, ND, former PWUD)

### 3.3 Social Environment

Factors related to the social environment were also listed as crucial elements in considering someone’s overall overdose risk exposure, again occurring at both micro and macro levels with some overlapping in both categories.

#### Micro risk factors

Participants discussed the emotional and psychological toll from witnessing, hearing of overdoses, and/or trying to revive someone. Frequently, participants discussed feelings of numbness from the frequency of these events and the trauma of witnessing deaths or near-deaths. Sometimes trauma manifested in indifference or distancing from others as a defense mechanism or difficulty grieving and connecting with the pain of loss:

It just like, I guess it’s like once you see it so much, it just…It’s just some everyday thing. There’s another one. There’s another one, and it’s sad but that’s the reality of it.

(457, Both)

It was not uncommon for participants to have lost a family member or loved one to overdose. Often, grief and trauma were cited as circumstances that lead to substance use in the first place. Many felt that if people had no desire to consume drugs in the first place, then substance dependence would not be an issue:

If people want to escape, yes….So, let’s find nothing -- let’s make the world so pleasant you want to not escape it. What can you do. You know what I mean? That’s a harder task than to drug use itself.

(398, Both)

For many participants, however, an important consideration was the fact that a contributor to risk is the enjoyment of using drugs and the relief they bring amidst challenging circumstances. One person described the enjoyable feeling of being high as a contributor to overdose risk:

It’s like the best rush I’ve ever had in my life…You know how it used to be when you was a kid and it was snowing outside. This is the only thing that I can pretty much get close, try to get close to it [inaudible], real cold outside, then your mother, you father bring you back from like an outing at night when it’s cold and you’re trying to get up in the car [inaudible], you just want to stay in that car and sleep. That’s all it’s like [inaudible]. But then I be saying to myself what if it ain’t really like that?

(626, Both)

Another social risk that numerous participants raised was the idea described as “greed” which held different meanings to participants, for instance: not knowing one’s tolerance level or pushing past it, using more than necessary, or not knowing one’s proper dosage. Not sharing resources and information with peers was perceived as selfish. A few participants reported wanting to get high immediately after having an overdose reversed:

They just take you to the hospital and then you go in there and I was just ready to get back out and go get high.

(626, Both)

One concern raised was the lack of support around intentional overdose. One person described an experience with a fellow treatment group participant:

A lot of people call her stupid, like give it up, you know, maybe go in-patient. Even the counselor’s like I don’t even want to hear it, you know, like we don’t want to hear it, you know. But, you know, she really needs to get, I think she’s more suicidal or looking for attention, because she knows what happens every time she gets high, she’s going to overdose.

(681, PWUD)

#### Macro risk factors

Within communities, there were significant risk factors related to existing power dynamics and social structures such as the constant policing of PWUD and systems of white supremacy that led people to feel unsafe. Some participants spoke about fears of calling police for an overdose. Immigrants may be afraid to call the police for fear of deportation. When called, police often failed to effectively respond to overdoses. In one incident reported by several participants, police neglected to administer naloxone to someone who was overdosing. There was a fear of stigma from police involving substance use:

When things like that happen, the last thing you want to do is involve the police. It’s not just that moment, either, it’s like a week later when they drive past you. Now all of a sudden, you’re affiliated with an illegal activity and that won’t go away.

(741, PWUD)

Some participants shared experiences of overdose risk related to observable bias and discrimination. One participant’s father’s death was due to a lack of easily accessible medications for opioid use disorder (MOUD). The participant felt that the lack of effective interventions was rooted in discrimination against certain groups (in this example, based on race) who use drugs:

Unfortunately, if he would had had some suboxone in jail, maybe he would have came out with some protection…My dad did not want to die that day. He wasn’t poor. He had resources. He went to programs. That tolerance dropped. There was no protection for my dad, none.

(489, ND)

#### Overlapping micro and macro risk factors

Many people shared that overdose risk was directly related to the amount of education the individual had received on harm reduction practices, such as how to use naloxone and regulating the quantity of substances used. These and related comments spoke to the micro-level role of the individual in responding to overdose, as well as systemic solutions regarding education and destigmatization efforts of both substance use and mental health.

Intergenerational mental illness, emotional distress, and internalized anger were related to substance use, as well as internalized stigma and shame. Interpersonal stigma could prevent one from seeking mental health treatment. These challenges were the norm for PWUD:

I haven’t met a person that’s doing drugs that say, “Yo, I just want to do it. Because I’m doing this because I feel like doing it. Because I got nothing else better to do.” They do it because they have all that, some physical, mental disability.

(116, PWUD)

#### Proposed solutions to social environment risk factors

Participants spoke about the need for harm reduction and drug use prevention education. Several participants wished for education with an emphasis on abstinence. Others suggested a focus on the negative consequences of substance use, particularly for children and youth. A related subject was the need for effective mental health and substance use treatment services. These programs also need to be a good “fit” for the individual by addressing the needs that bring that person to treatment:

“Some groups are snobs even if you’re on methadone…Willpower, you know, and they’re not, I don’t think they’re really for the Narcan. I think they would, you know, actually turn they nose up at it.“

(681, PWUD)

These services also needed to address the whole person and encompass the impacts of intergenerational trauma as a contributor to overdose. In the excerpt below, the participant shared ways to counteract structural barriers that involved improvement of relationships and community infrastructure, such as broad grassroots organizing and open, non-stigmatizing discussions around issues impacting PWUD:

It’s too much talking and only a few that’s really doing the work. And their work is on the ground. The work is grassroots. We need to leave our computers and desks and nice jackets home and go out there with the community that’s suffering.

(489, ND)

### 3.4 Economic Environment

While less commonly cited, economic factors were still a considerable concern for overdose risk occurring at both the micro and macro levels. For some, wealth and income were directly tied with possible overdose risk, in ways that manifested on both a micro and a macro level.

#### Micro risk factors

At the micro level, one example shared by an interviewee reported that access to money could lead to relapse. To prevent this, they gave someone their debit card to hold. However, lack of financial resources was also a significant stressor that could lead one to relapse to cope.

#### Macro risk factors

Others highlighted institutional barriers to treatment and recovery which included insufficient treatment options and resources for recovery. The government was seen as uninvested in contributing to a lack of supportive resources, including substance use treatment resources in the community. Similarly, a lack of housing, affordable healthcare, and employment opportunities were seen as ways that PWUD remain in a cycle of poverty fueled largely by a profit-driven economic system:

I think people are forced into bad situations. They lose access to housing. They lose access to steady food sources, medical care, a lot of people’s needs are just not being met, and government austerity is really what just makes this sort of survivalist approach to drug use necessary.

(828, ND)

Factors related to the drug market were additionally of great concern, such as the role of drug dealers who often lacked necessary knowledge on providing a safe supply. Many participants were concerned with the deregulation of the drug market, as it allowed drug dealers operating illegally to make profit-driven choices with no oversight, contributing to more dangerous drugs and higher overdose risk.

Participants also observed that over-monitoring of prescription drugs limits necessary access to pain medication. There were concerns related to “big pharma” and an over-prescribing of prescription pain medications driven by the pursuit of profit, leading to the modern prescription drug crisis:

“And I think they need to stop with these crooked doctors prescribing people pills.”

(411, PWUD).

Finally, interviewees remarked on the increased presence of fentanyl as a driver of overdoses, particularly in communities that are already marginalized in other ways.

#### Overlapping micro and macro risk factors

Income inequality and a sense that the drug market preyed upon PWUD to make money for those with more power was a frequently cited economic concern.

We don’t care, man, about people. We just don’t. And those who do care about people, we’re made to feel bad about it, and so yeah, I mean, I can point to capitalism. I can point to white supremacy.

(586, ND)

#### Proposed solutions to economic environment risk factors

Several solutions were shared by participants on how these problems might be curbed. To address the impact of fentanyl, participants stated a need to know its source, stop the supply, and address the increase of fentanyl during the COVID-19 pandemic. Additionally, measures should be taken to address the role of prescription drugs in fueling the drug crisis:

They need to stop prescribing people Percs…They gave it to my mother and she couldn’t take it, she got sick, cause its addicting.

(411, PWUD)

One person remarked that not having adequate access to resources to meet one’s basic needs drives many people to use substances, and they proposed that a better overall standard of living would lead to lower need for substance use as a coping mechanism:

And then just having a livable I guess standard of living that doesn’t drive us to seek out potentially -- I don’t know if that’s really the right thing to say, just having a better standard of living.

(828, ND)

### 3.5 Policy Environment

Finally, study participants referred only to overlapping micro and macro elements at the policy level that could increase their propensity for overdose risk, and offered some suggestions for resolution to those policy-level risk factors.

#### Overlapping micro and macro risk factors

Some policy risk factors were related to the scarcity of harm reduction service providers, the lack of naloxone availability, the inaccessibility of MOUD, and the dearth of widespread prevention education. Participants also highlighted the consequences of the current limitations on prescription pain medication that resulted in abruptly discontinuing patients and drove them into the unsafe illicit drug market. Criminalization of drugs and people who use drugs was a major concern for participants of the study.

One participant stated that the criminal legal system doesn’t care about PWUD and that drug courts are punitive and ineffective.

So we going to put you on a Drug Court for five years and all you had was two bags of dope. I mean, how does that add up? It don’t add up. So now I’m stuck here for five years and I’m still an addict for two bags of dope.

(457, Both)

#### Proposed solutions to policy environment risk factors

A need was raised for improved long-term recovery services and alternatives to 12-step programs. Another person noted that the emphasis on abstinence as the prevailing treatment modality was a barrier to receiving MOUD and naloxone. Policies related to more expansive definitions of treatment and recovery were needed to reduce overdose risk:

It’s like look man, if you want treatment you could go to a 12-step program, but what if I needed that 13th step, or 27th step or 28th? You know what I mean? Because I faked it through the whole process, the whole 12 steps, man, and I really haven’t gotten what I needed.

(586, ND)

Some wanted to broadly alter substance use by regulating and decriminalizing substances to make them safer at the societal level:

*Regulated -- legalized drugs. Make them safe. That’s the only change that could be made. Make them usable, just like to make the lowest risk possible for being fatal. That’s the only thing you can do. (398, Both)* Safe consumption was another commonly proposed solution, including overdose prevention centers where people could use drugs safely.

## 4. CONCLUSION

Findings from the present study show that factors from all four categories of Rhodes’ risk environment (4) are at play in impacting the likelihood of overdose for PWUD in New Jersey. Individuals included in this study identified elements of the physical environment, social environment, economic environment and policy environment as factors contributing to their risk of overdose. Physically, participants felt a lack of space to use drugs safely and a frustration that their physical environment felt like it increased their risk of overdose. Socially, participants noticed a lack of education on overdose risks and a culture that forced substance use into the shadows and perpetuated greater risk of overdose. Economically, a lack of affordable housing and income inequality were major contributors to risk. And finally, the policy environment was a barrier to harm reduction and limited access to resources to use substances safely, leading to a greater risk of dangerous outcomes. These risks were found to occur on micro and macro scales, implying necessary intervention at both the individual and community levels.

At the macro level, systemic and institutional risks and stigma towards PWUD were named as major considerations in the risk environment. On the micro level, emergent themes included mental health as a risk factor, substance use behaviors, the process of pursuing treatment and recovery, and the role of apathy and histories of trauma in contributing to risk of overdose. These considerations exemplify the major finding of the risk environment framework that overdose risk is contingent upon the social context (4). Even when risks emerge at the individual level, they are situationally determined and demand a community-level response.

The findings also demonstrate the distinction between *susceptibility* and *vulnerability* to risk that Rhodes (4) outlines in his initial risk environment framework, and further contextualizes these concepts in New Jersey—an example of a diverse, urban and relatively liberal policy environment. Risk factors can contribute to a community’s susceptibility to overdose (or the rate at which overdose risk materializes) as well as its vulnerability (the likelihood that a risk factor will have dangerous impacts). Susceptibility to overdose is increased in New Jersey in part because of the policy environment, where a decades-long municipal ordinance meant overdose prevention resources were only available in municipalities whose governing boards actively approved harm reduction centers (18). Vulnerability to the risk of overdose is demonstrated by the economic disenfranchisement within the two communities (19).

In addition to a call for increased research on the risk environment framework among racial/ethnic minority communities, related studies on the risk environment framework have advocated for gathering and employing recommendations from people with lived experience to inform policy and practice interventions to address environmental risk factors (20). This study responds specifically to this reported lack of research on risk environments among PWUD in racial/ethnic minority populations (21). Research advocating for centering the voices of PWUD in policy, program, and practice design is increasingly common, and the risk environment framework is commonly used as a starting point for those findings (22, 23). In the next section, specific policy recommendations and procedures for addressing environmental risk factors will be outlined.

This study is not without limitations. Utilizing the framework proposed by Padgett et al. (24) for evaluating the credibility, transferability, and trustworthiness of qualitative research, this study employed rigorous efforts to ensure credibility and trustworthiness of findings by having a diverse study team, thorough audit trail, and a CBPR approach that included member checking and communal data analysis approach. However, the transferability of the findings may be limited to comparable geographic regions, but due to the nature of the risk environment framework this need for localized efforts is unavoidable. It is also possible that the sample’s limitation to service-involved PWUD excluded the perspectives of non-service involved individuals who may have provided an important unique perspective on the local risk environment.

## 5. POLICY IMPLICATIONS

Original applications of the risk environment framework propose responding to the reality of a multi-dimensional risk environment with a social epidemiological approach that recognizes drug-related harm as a public health concern with inextricable social underpinnings (4). In Rhodes’ proposal, interventions towards drug-related harm reduction (specifically HIV infection) would include social efforts that create an *enabling environment* for harm reduction interventions. An enabling environment is one in which local context is hospitable to harm reduction policies and practices, and those practices are implemented and responsive to changing community needs. They are formulated in conversation with people who use drugs and require the consistent involvement of policy makers, practitioners, and community members to ensure quality and consistency of efforts. An enabling environment improves the political-economic circumstances of PWUD, shapes the physical environment based on the logics of everyday habits contributing to overdose risk, and creates space for healing and transformation especially in communities where overdose risk is most acutely present. Studies on enabling environments have demonstrated the impact that peer support, stigma reduction, and compassionate service design can have on increasing capacity for harm reduction and reducing overdose risk (25, 26).

This paper’s proposed interventions and policy considerations are derived from both the risk environment literature and directly from conversations with PWUD during this study. The risk environment framework advocates evaluating existing programs (such as syringe exchange programs [SSP] or Opioid Overdose Education and Naloxone Distribution [OEND] programs) through the risk environment framework to determine factors driving susceptibility and vulnerability to harm within the context of that intervention. The framework then inquires how those factors can be predicted, prevented, and reduced in that specific environment. An example of this process using the findings obtained from PWUD in this study could involve an SSP or OEND program considering the risks of using alone and need for harm reduction education, and service providers could place emphasis on educating program participants on safer use practices in addition to distributing supplies (27). Another example of a risk environment-informed intervention is acknowledging the role of mental health and trauma as major contributors to overdose risk and providing related resources to address these root causes of substance use (28). Overall, service providers should prioritize relationship-building and an ongoing process of maintaining awareness of the local risk environment to instruct their efforts, as well as providing increased funding for known solutions, equity of access and opportunity, and accessibility of resources to all who need them.

This process should also be implemented on the macro level, so that community-level policies and individual practice decisions are both informed by the local risk environment. At the policy level, risk environment-informed decision-making could be understood as decriminalization of drug use (29), increased federal regulation and oversight of the drug market without further criminalization of PWUD (30), and policy improvements regarding limits on legal prescription drugs for pain management that make people turn to dangerous, illegal sources (31). It should also consider economic factors to inform policies supporting basic income, accessible housing, and other basic standards of living that have a demonstrated strong correlation with increased overdose risk (32).

Ultimately, policies directed towards the well-being of PWUD should be aware of the local factors contributing to risk and avoid increasing these harms, emphasizing harm reduction efforts that are informed by a harm reduction philosophy. Policies that do not take these ongoing risk factors into account are likely to increase overdose risk and lead to greater death. The findings of this study endorse the relevance of the risk environment framework as a starting point for assessing overdose risk when formulating policy and practice decisions impacting PWUD.

## Figures and Tables

**Table 1:** Interview Sample Descriptive Statistics

Descriptor	n	%
**Gender**		
Man	18	60.0%
Woman	10	33.3%
Non-Binary	2	6.7%
**Race/Ethnicity**		
Asian/Pacific Islander, Hispanic/Latinx, & Other*	3	9.9%
Black/African American	24	80.0%
White	3	10.0%
**Participation Category**		
People Who Use Drugs (PWUD)	8	26.7%
Naloxone Distributors (ND)	3	10.0%
Both	19	63.3%

* Categories collapsed to preserve participant anonymity

**Table 2.** Presentation of Major Themes of Overdose Risk Environment, Illustrative Quotes & Proposed Interventions

Major Themes	Minor Themes	Illustrative Quotes	Proposed Interventions
Physical Environment	Micro Risks • Using drugs alone • Being unable to distance oneself from drug use Macro Risks • Community hot spots • Inadequate shelter • Systematic resource redistribution Micro/Macro Risks • Stigma & taboo • Exiting treatment/leaving a setting without substance use	So if an individual’s mandated, you know, into treatment or into detox, or even incarceration, right, when they come out, if they relapse, they’re not going to start back at the beginning again. So they’re going to, you know, relapse and use up that last amount. But they don’t realize their tolerance level has dropped. (718, ND) You go downtown. A lot of them be out there overdosing. They be, they be on, they be on a tilt out there, leaning…They said, on my block, they said somebody was there dead for 3 days and they just left them on the steps off of overdosing. (199, PWUD) Again, you know, there are counties, there are towns where we say hey, can we come in, and they’re like no thank you, you know, we don’t have a drug problem here. And it’s not true, right? (718, ND) I just wish they could get off the streets and do something better for themselves, ‘cause there’s a lot of talented people in New Brunswick that’s on heroin. That’s very smart. They go to jail. They can draw. They can write poems. They can do this. But then when you see them on the streets… I wish they find shelter. (200) You have people that pass out free needles and like that, but they don’t really like, even people that come down here, but that’s only for, only to make it look good for like, nah. Do that shit faithfully. Like go out there every week and like, you know like come on? Like come give them a checkup, like people are out there, like they out there. (111, PWUD)	• Overdose prevention centers/safe consumption sites • Increased treatment and other resource access • Support for individuals exiting incarceration • Geographically targeted harm reduction interventions
Social Environment	Micro Risks • Grief and trauma of experiencing, responding, or bearing witness to overdose • “Greed” Macro Risks • Racism & white supremacy • Criminalization of PWUD Micro/Macro Risks • Lack of comprehensive harm reduction/safe substance use education • Lack of mental health and substance use treatment • Drugs as treatment	When things like that happen, the last thing you want to do is involve the police. It’s not just that moment, either, it’s like a week later when they drive past you. Now all of a sudden, you’re affiliated with an illegal activity and that won’t go away. Sad but true, it don’t. (741, PWUD) In my experience with people I’ve lost, they had to hide it…And we know that’s how people die…People die in those shadows, you know? (520, ND, formerly PWUD) I haven’t met a person that’s doing drugs that say, “Yo, I just want to do it. Because I’m doing this because I feel like doing it. Because I got nothing else better to do.” They do it because they have all that, some physical, mental disability. (116, PWUD) It just like, I guess it’s like once you see [overdose] so much, it just…It’s just some everyday thing. There’s another one. There’s another one, and it’s sad but that’s the reality of it. (457, Both) If people want to escape, yes….So, let’s find nothing - - let’s make the world so pleasant you want to not escape it. What can you do. You know what I mean? That’s a harder task than to drug use itself. (398, Both) Because if someone has, you know, gone into diabetic shock, everybody’s going to call 911 and say oh my gosh what can we do. If someone overdoses, that’s not the same conversation, that’s not the same 911 call. (718, ND)	• Comprehensive education for PWUD, families, and youth on substance use, including abstinence • Increased mental health and trauma treatment availability • Countering white supremacy and systematic racism
Economic Environment	Micro Risks • Lack of OR access to finances Macro Risks • Unregulated drug markets • Income inequality, greed, & pharmaceutical company benefit • Lack of government investment and priority in the needs of PWUD • Fentanyl	Unfortunately, if he would had had some suboxone in jail, maybe he would have came out with some protection. That’s the only thing I could say that would have maybe turned that scenario around, MAT. The Narcan being available there for him would have been helpful, too. Maybe I would still have my dad on earth, you know? (489, ND) I was just speaking with a colleague from Puerto Rico who was like isn’t there Housing First that should deal with that? And I was like New Jersey on paper, yes, but not really. (520, ND, formerly PWUD) We don’t call them harm reduction. We call them survival, and we just think of it as our lifestyle and just that if we listen more to people like that, like all the things that people are just discovering this year could have been discovered 50 years ago. (520, ND, formerly PWUD) Those spaces where you can just be out loud and you can say I’m using drugs today, can I hang out here, because I don’t have anywhere else to be. (520, ND, formerly PWUD) I think people are forced into bad situations. They lose access to housing. They lose access to steady food sources, medical care, a lot of people’s needs are just not being met, and government austerity is really what just makes this sort of survivalist approach to drug use necessary. (828, ND)	• Increased financial resources & government benefits availability • Pharmaceutical company regulation • Safe supply
Policy Environment	Micro/Macro Risks • Local municipal ordinance • Criminalization of drug use • Medications for opioid use disorder (MOUD) regulation	Regulated -- legalized drugs. Make them safe. That’s the only change that could be made. Make them usable, just like to make the lowest risk possible for being fatal. (398, Both) So we going to put you on a Drug Court for five years and all you had was two bags of dope. I mean, how does that add up? It don’t add up. So now I’m stuck here for five years and I’m still an addict for two bags of dope. (457, Both) It’s like look man, if you want treatment you could go to a 12-step program, but what if I needed that 13th step, or 27th step or 28th? You know what I mean? Because I faked it through the whole process, the whole 12 steps, man, and I really haven’t gotten what I needed. (586, ND)	• Removal of barriers to establishing harm reduction centers • Decriminalization of drug use/people who use drugs • Safe supply • Reforming drug court • Increased MOUD availability

## Data Availability

Data from this study are not publicly available due to reasons of sensitivity.

## Abbreviations

PWUD: People who use drugs
OEND: Overdose Education and Naloxone Distribution
CBPR: Community-based participatory research
MOUD: Medications for Opioid Use Disorder
ND: Naloxone Distributor
Percs: Percocet
